# Integrating Psychodermatology and Platelet-Rich Plasma Therapy in Various Dermatological Conditions: A Narrative Review

**DOI:** 10.7759/cureus.95055

**Published:** 2025-10-21

**Authors:** Ansa Bakhtawar, Ayesha Mujeb, Isra Meraj, Bashir Imam, Varthini Karvannan, Paola Chavez, Zain Ul Abideen, Lalain Masood, Zoya Morani

**Affiliations:** 1 Medicine, Basildon University Hospital, Basildon, GBR; 2 Medicine, University of Glasgow, Glasgow, GBR; 3 General Practice, Nanavati-Max Super Speciality Hospital, Mumbai, IND; 4 Internal Medicine, Jackson Park Hospital and Medical Center, Chicago, USA; 5 Internal Medicine, King's College Hospital, London, GBR; 6 Plastic Surgery, Hospital Maria Auxiliadora, Lima, PER; 7 Internal Medicine, Lahore General Hospital, Lahore, PAK; 8 Dermatology, Bahria University Medical and Dental College, Karachi, PAK; 9 Family Medicine, Washington University of Health and Science, San Pedro, BLZ

**Keywords:** integrative dermatology, prp and its uses, prp and psychodermatology, psychological outcomes of prp therapy, psychology and dermatology

## Abstract

Traditional dermatological care often prioritizes visible symptoms, while under-recognizing psychological distress and overlooking emerging regenerative therapies. This narrative review examines two adjunctive approaches: psychodermatology, which addresses the bidirectional relationship between psychological health and skin disease, and platelet-rich plasma (PRP), a minimally invasive regenerative treatment with demonstrated efficacy in acne scarring, alopecia, melasma, vitiligo, and wounds through growth factor-mediated repair. Psychodermatology, underpinned by the neuro-immuno-cutaneous system (NICS), highlights how psychological stress can exacerbate dermatological disease and impair quality of life (QoL). The mechanisms and clinical applications of both modalities are explored, alongside their potential to enhance patient-centered care in chronic skin conditions (CSCs). Integrating PRP and psychodermatology into practice may improve both clinical outcomes and QoL, highlighting the need for multidisciplinary, patient-centered care.

## Introduction and background

Despite advances in topical and systemic therapies, conventional dermatological treatments often prioritize visible symptoms, while overlooking emotional distress, treatment resistance, adverse effects, and recurrence [1]. Chronic skin conditions (CSCs), such as acne vulgaris and androgenetic alopecia (AGA), affect both physical appearance and psychological well-being. In this context, platelet-rich plasma (PRP) and psychodermatology have emerged as adjunctive approaches that address both biological mechanisms and psychosocial dimensions of skin disease.

PRP, developed in the 1970s for hematological use, has since been adopted in dentistry, orthopedics, and dermatology for its regenerative potential [2]. Rich in autologous growth factors - including platelet-derived growth factor (PDGF), vascular endothelial growth factor (VEGF), and transforming growth factor beta (TGF-β) - PRP promotes angiogenesis, fibroblast proliferation, and collagen remodeling [2,3]. It has shown clinical benefit in treating acne scarring, AGA, melasma, and chronic wounds, offering a minimally invasive and well-tolerated adjunctive therapy in dermatological practice [2]. Table 1 outlines the mechanism of action of PRP factors and their dermatological relevance.

**Table 1:** PRP Components and Their Dermatological Relevance Table credit: [4-9], created via Microsoft Word (Microsoft® Corp., Redmond, WA, USA) by Bashir Imam. VEGF: Vascular Endothelial Growth Factor; PDGF: Platelet-Derived Growth Factor; EGF: Epidermal Growth Factor; TGF-β: Transforming Growth Factor Beta; PRP: Platelet-Rich Plasma

Elements of PRP	Mechanism of Action	Dermatological Relevance
VEGF	Increases neovascularization and blood supply	Helpful androgenetic alopecia and chronic scarring
PDGF	Promotes fibroblast and smooth muscle proliferation	Helpful for wound healing, skin rejuvenation, and acne scars
EGF	Activates keratinocyte and fibroblast proliferation	Used to improve skin texture and rejuvenation; helpful for acne scars.
TGF-β	Promotes collagen synthesis and production; reduce inflammation	Helpful for scar remodeling and wrinkle reduction; post-procedure healing.

Psychodermatology, meanwhile, examines the bidirectional relationship between psychological health and skin disease. Psychological stress can precipitate or aggravate dermatological conditions, while visible skin disease can contribute to anxiety, depression, and low self-esteem [1,10]. Up to 30% of CSCs have a psychiatric component, with particularly high psychological burden in conditions like acne, atopic dermatitis, psoriasis, vitiligo, and herpesvirus infections [10]. Mental health comorbidities may impair adherence and exacerbate disease progression, whereas integrated psychosocial care improves compliance, reduces maladaptive behaviors (e.g., skin picking), and enhances quality of life (QoL) [11].

This review aims to explore the evolving role of PRP and psychodermatology as adjunctive therapeutic approaches in dermatology, highlighting their mechanisms, clinical applications, and potential to advance patient-centered, integrative dermatological care. A structured PubMed search (2015-2025), using terms including “PRP in dermatology” and “psychotherapy in dermatology,” and applying sidebar filters and examining references in selected articles, informed this analysis. 

## Review

PRP and its uses in dermatological conditions

PRP has emerged as an adjunctive or alternative modality to conventional therapies in the management of acne vulgaris [12]. Comparative studies indicate that PRP offers superior improvement in both active acne lesions and atrophic scarring compared with topical erythromycin 2% and microneedling alone, respectively [12,13]. Additionally, PRP may reduce dependence on long-term antibiotic regimens and isotretinoin, mitigating risks such as antimicrobial resistance and teratogenicity [12]. Combination therapies involving PRP and microneedling demonstrate significantly greater reductions in scar depth, enhanced skin texture, and higher patient satisfaction than either modality alone [13]. A clinical trial further reported that the addition of PRP and Yifu to ultra-pulsed CO₂ laser therapy increased the overall response rate (81.43%) compared to laser monotherapy (70.00%) [14].

Beyond acne, PRP has been evaluated in the management of melasma, a chronic pigmentary disorder with psychological and cosmetic implications. While conventional therapies - such as hydroquinone, azelaic acid, kojic acid, glycolic acid, tranexamic acid, and antioxidants (e.g., vitamins C and E) - remain the standard of care [4], PRP has shown comparable or superior efficacy. In a split-face trial, PRP outperformed intradermal tranexamic acid in reducing melasma severity [15]. Additionally, when used adjunctively with hydroquinone, PRP resulted in 51%-75% improvement in most patients, versus just 3.3% in the hydroquinone-only group [4].

PRP also shows promise in the treatment of alopecia, particularly in AGA and immune-mediated forms such as alopecia areata (AA) and primary cicatricial alopecia. In AGA, dihydrotestosterone (DHT)-driven miniaturization of follicles limits the efficacy of current pharmacologic options such as minoxidil and finasteride [5]. PRP delivers growth factors that activate anti-apoptotic and proliferative pathways (e.g., ERK, Akt, Wnt/β‑catenin), promoting follicular regeneration and angiogenesis [6]. Studies combining PRP with minoxidil report enhanced outcomes compared to monotherapy [16]. Notably, PRP monotherapy has yielded mixed results - one study reported that 72.2% of patients showed no clinical change at three months, with only 33.3% showing notable improvement at six months [16]. These findings highlight the need for further mechanistic studies and standardization of protocols, as heterogeneity in preparation remains a major barrier to clinical translation [5].

In vitiligo, an autoimmune disorder characterized by melanocyte destruction, PRP has emerged as a potential adjunctive therapy. The primary treatment goal is re-pigmentation through melanocyte stimulation [7]. While conventional approaches include corticosteroids, calcineurin inhibitors, systemic immunosuppressants, Janus kinase inhibitors, and narrowband ultraviolet B (NB-UVB) phototherapy [17], PRP may enhance therapeutic outcomes by promoting fibroblast-derived growth factors (FGFs) and chemokines that support melanocyte migration and proliferation [17]. Combined protocols involving PRP and phototherapy (e.g., NB-UVB or excimer laser) appear to yield superior outcomes in terms of pigmentation and patient satisfaction [17].

PRP has also been applied in chronic wound management, including diabetic foot ulcers and venous leg ulcers, which pose a significant clinical burden due to delayed healing and recurrences [18]. PRP reduces wound size, with better responses observed in venous ulcers than diabetic ulcers, likely due to anatomical and vascular differences [18]. Platelet-rich fibrin (PRF), a second-generation platelet concentrate, is produced without anticoagulants, allowing natural fibrin clot formation and slower, sustained release of growth factors. Comparative trials suggest similar clinical efficacy and safety for PRP and PRF, with wound size reductions of 46.1%-73.8% for PRP and 44.8%-71.4% for PRF [8,19]. Advanced platelet-rich fibrin (A-PRF) offers a prolonged release of growth factors over around 10 days, whereas PRP releases its growth factors within 15-60 minutes, supporting its use in scenarios requiring rapid tissue repair [20]. This rapid release profile supports PRP’s utility in clinical scenarios requiring accelerated tissue repair and early wound healing.

**Figure 1:**
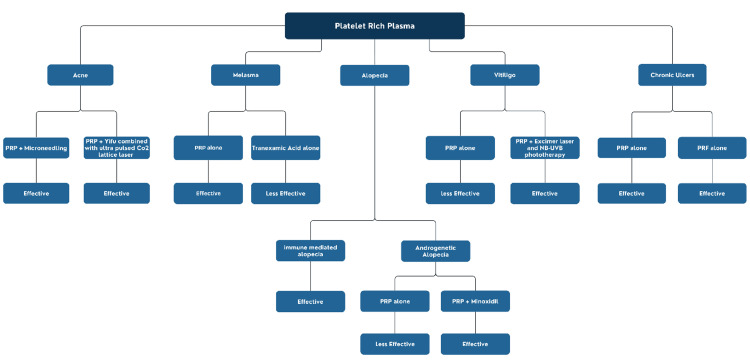
Uses of Platelet Rich Plasma in Dermatology Image credit: [4-8,12-19], created via Canva (Canva Pty Ltd., Sydney, Australia) by Ansa Bakhtawar PRP: Platelet-Rich Plasma; PRF: Platelet-Rich Fibrin; NB-UVB: Narrowband Ultraviolet B

In patients with diabetes, PRP has accelerated ulcer healing and reduced healing time as an adjunct to standard care [21,22]. Overall, PRP demonstrates efficacy across multiple dermatological conditions, but protocol variability and evidence quality remain limitations, reinforcing the need for standardized preparation and further high-quality trials. Figure 1 highlights the uses of PRP in dermatology.

Role of PRP and psychodermatology in alopecia

AGA and AA are among the most common causes of chronic non-scarring hair loss, frequently associated with significant psychosocial distress and diminished QoL [23,24]. AGA is driven by the enzymatic conversion of testosterone to DHT, which induces progressive miniaturization of affected hair follicles, shortening the anagen phase and transforming terminal hairs into thin, vellus hairs [23]. In contrast, AA is an autoimmune condition marked by collapse of immune privilege in hair follicles, often triggered or exacerbated by chronic stress and neuroimmune dysregulation involving corticotropin-releasing hormone (CRH), substance P, and the Th1/Th17 axis [9].

Patients with AA exhibit higher rates of depression and anxiety, contributing to increased time taken off employment [24]. In AGA and telogen effluvium (TE), elevated proinflammatory cytokines (e.g., IL-1 and TNF-α) and oxidative stress are implicated in the transition to the telogen phase and follicular regression [9]. Psychocutaneous disorders, such as trichotillomania (TTM) and body dysmorphic disorder (BDD), involve compulsive hair manipulation and distorted body image, often requiring integrated dermatological and psychiatric care. Affective disorders are also linked to reduced brain-derived neurotrophic factor (BDNF), which may impair dermal papilla function and disrupt the hair cycle [9]. These findings highlight the importance of a biopsychosocial framework, including stress management, psychotropic medication review, and integrated care models [9].

PRP has emerged as an adjunctive therapy for AGA, providing a regenerative approach through a rich concentration of autologous growth factors that influence key hair structures, including the bulge region, dermal papilla, and perifollicular vasculature, promoting follicular survival and growth via activation of the ERK, Akt, and Wnt/β‑catenin pathways [5,25]. Despite promising results, heterogeneity in PRP preparation protocols and outcome measures remains a major barrier to clinical translation. The 2018 S3 guideline rated PRP as Level 3 evidence for AGA and female pattern hair loss (FPHL), due to methodological limitations [25]. More recent reviews have upgraded PRP to Level 1a evidence, acknowledging consistent efficacy but still recommending harmonization of protocols [25].

Meta-analyses of 16 RCTs and 13 split-scalp studies indicate increased hair count and diameter, although not always statistically significant [26,27]. PRP has also improved hair texture, density, and seborrhea, particularly in early-stage alopecia [28,29]. However, only 26% of the 23 RCTs evaluated were considered high quality [25]. A 2024 meta-analysis demonstrated that combining PRP with 5% topical minoxidil improved hair density significantly at one, three, five, and six months, with no added side effects [30]. Although the certainty of evidence remains low, these consistent improvements support a synergistic model that integrates regenerative and pharmacologic therapy. 

Another RCT showed superior results with PRP and microneedling, particularly regarding hair shaft diameter and patient satisfaction [31]. A retrospective study on triple therapy (PRP, minoxidil, and microneedling) produced the most robust results, although it was limited by small sample sizes and PRP preparation variability [31]. A meta-analysis of 10 RCTs covering 555 treatment sites found a mean gain of 25 hairs/cm² but no significant improvement in hair diameter [32]. Subgroup analysis revealed higher density gains in male-only trials, likely due to androgen-dominant pathophysiology, higher scalp androgen receptor density, and more predictable follicular miniaturization - potentially enhancing responsiveness to PRP’s growth factor cascade [32,33].

**Figure 2:**
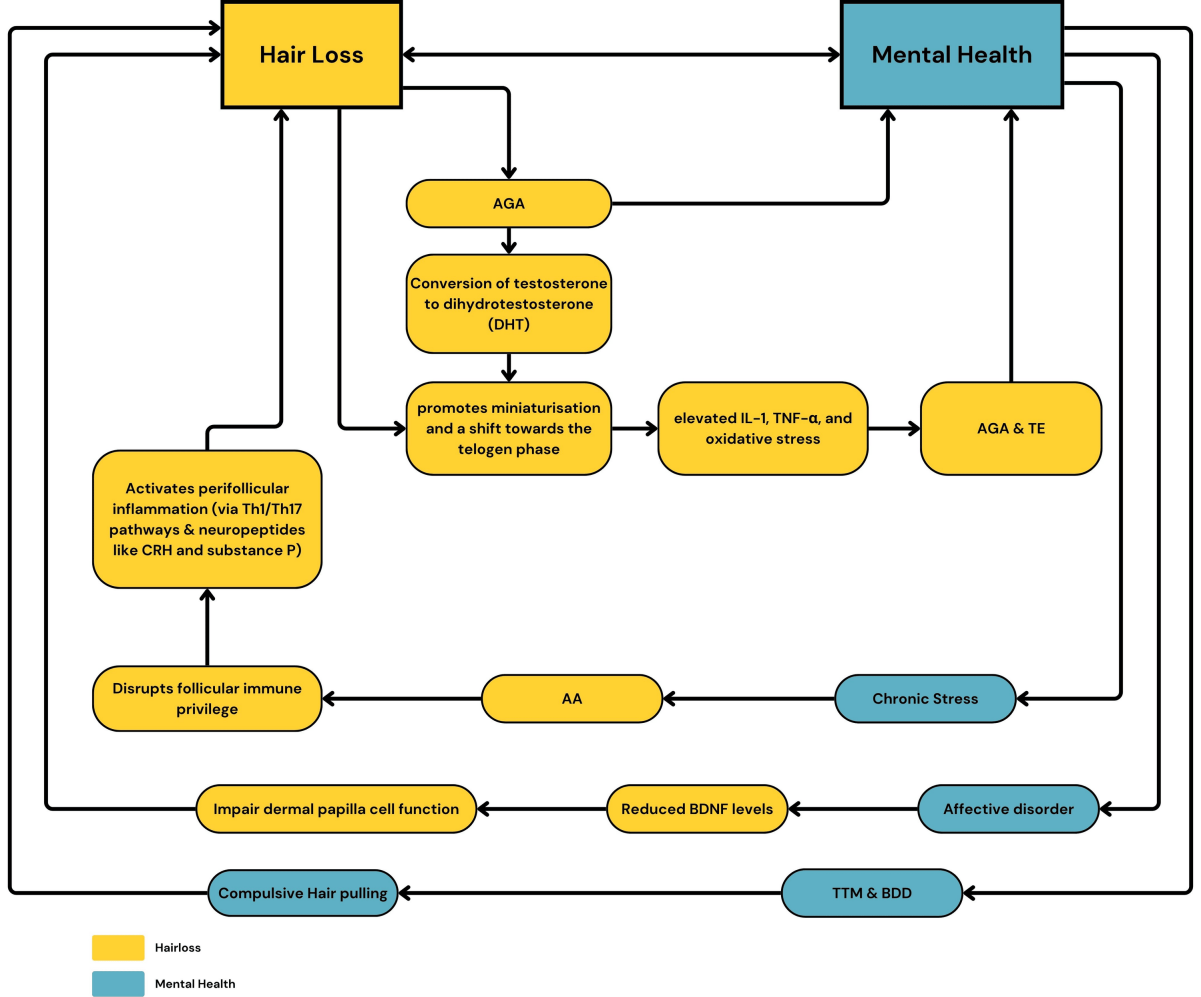
Relationship Between Mental Health and Hair loss Image credit: [9,23], created via Canva (Canva Pty Ltd., Sydney, Australia) by Ansa Bakhtawar. AGA: Androgenetic Alopecia; AA: Alopecia Areata; TE: Telogen Effluvium; TTM: Trichotillomania; BDD: Body Dysmorphic Disorder; CRH: Corticotropin-Releasing Hormone; BDNF: Brain-Derived Neurotrophic Factor

PRP is a low-risk, autologous treatment with growing evidence for hair restoration, particularly alongside topical agents and microneedling. Its integration within a psychodermatological care model provides dual benefits by addressing both the physiological and emotional burden of hair loss, optimizing clinical outcomes and QoL. Figure 2 outlines the link between hair loss and mental health.

Psychodermatology: mechanisms and clinical relevance

CSC, particularly when visible, imposes significant psychological, social, and economic burdens. They are associated with heightened self-consciousness, depression, anxiety, obsessive-compulsive symptoms, suicidal ideation, and fear of stigma - all of which negatively impact QoL and social participation [34,35]. Psychiatric comorbidity is estimated to affect 30%-60% of dermatological patients [36], yet remains under-recognized in routine practice. In the Netherlands, for example, dermatologists refer on average only eight patients per year for psychological treatment, with half never having referred a patient to a psychologist or psychosocial therapist [37]. Globally, up to one in three patients with skin disease experience undetected or untreated mental health symptoms [35].

**Figure 3:**
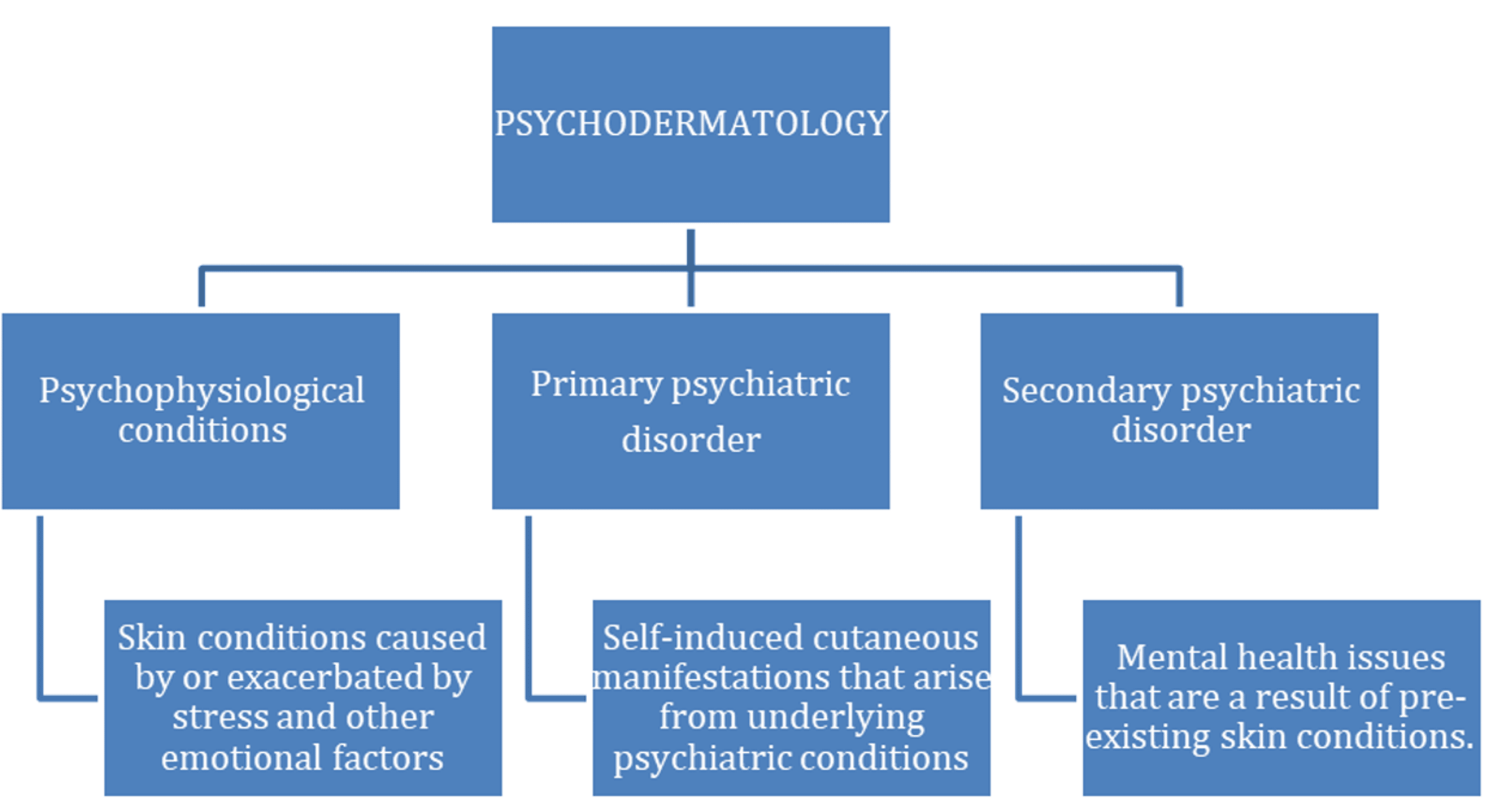
Classification of Psychodermatology Image credit: [36], created via Microsoft Word (Microsoft® Corp., Redmond, WA, USA) by Paola Chavez.

The connection between skin and psychological health is mediated not only by behavioral factors but also by overlapping neuroimmune pathways, including the hypothalamic-pituitary-adrenal (HPA) axis, neuropeptides such as substance P, and other mediators that drive both systemic inflammation and stress responses [38]. Psychodermatology examines these interactions through the neuro-immuno-cutaneous system (NICS) [36,39], recently expanded to the neuro-immuno-cutaneous-endocrine (NICE) model, which conceptualizes skin as a dynamic neuro-immuno-endocrine interface engaged in multidirectional homeostatic signaling [40]. Dysregulation of these systems contributes to both cutaneous and psychiatric manifestations, particularly in inflammatory dermatoses (e.g., eczema, psoriasis, acne, and rosacea) and pigmentary or scarring disorders (e.g., vitiligo, melasma, and keloids) [36,37]. Figure 3 illustrates the types of psychodermatology.

Dysregulation of these systems contributes to both cutaneous and psychiatric manifestations. In eczema, chronic sympathetic overactivity can trigger acetylcholine release from cutaneous autonomic fibers, provoking pruritus independent of external stressors [40]. In psoriasis, hyperactivation of the HPA axis may mediate the established relationship between psychological stress and disease severity [37]. Atopic dermatitis increases the risk of depression and suicidal ideation in younger populations [41,42]. Similar neuroendocrine-immune mechanisms are implicated across inflammatory, pigmentary, and scarring dermatoses [36].

Integrated care models are increasingly recognized as optimal for managing these complex interactions. Multiple psychotherapeutic modalities, including cognitive behavioral therapy (CBT), mindfulness-based interventions, and hypnotherapy, have demonstrated improvements in both psychological and dermatological outcomes [11,37,43]. Pharmacological interventions such as antipsychotics and anxiolytics may be required in some psychocutaneous disorders, but they necessitate careful monitoring for side effects, including sedation, weight gain, and adherence challenges [44]. Collaborative psychodermatology clinics uniting dermatologists, psychiatrists, and psychologists are particularly effective for disorders such as skin picking, TTM, BDD, and delusional infestation [45].

**Figure 4:**
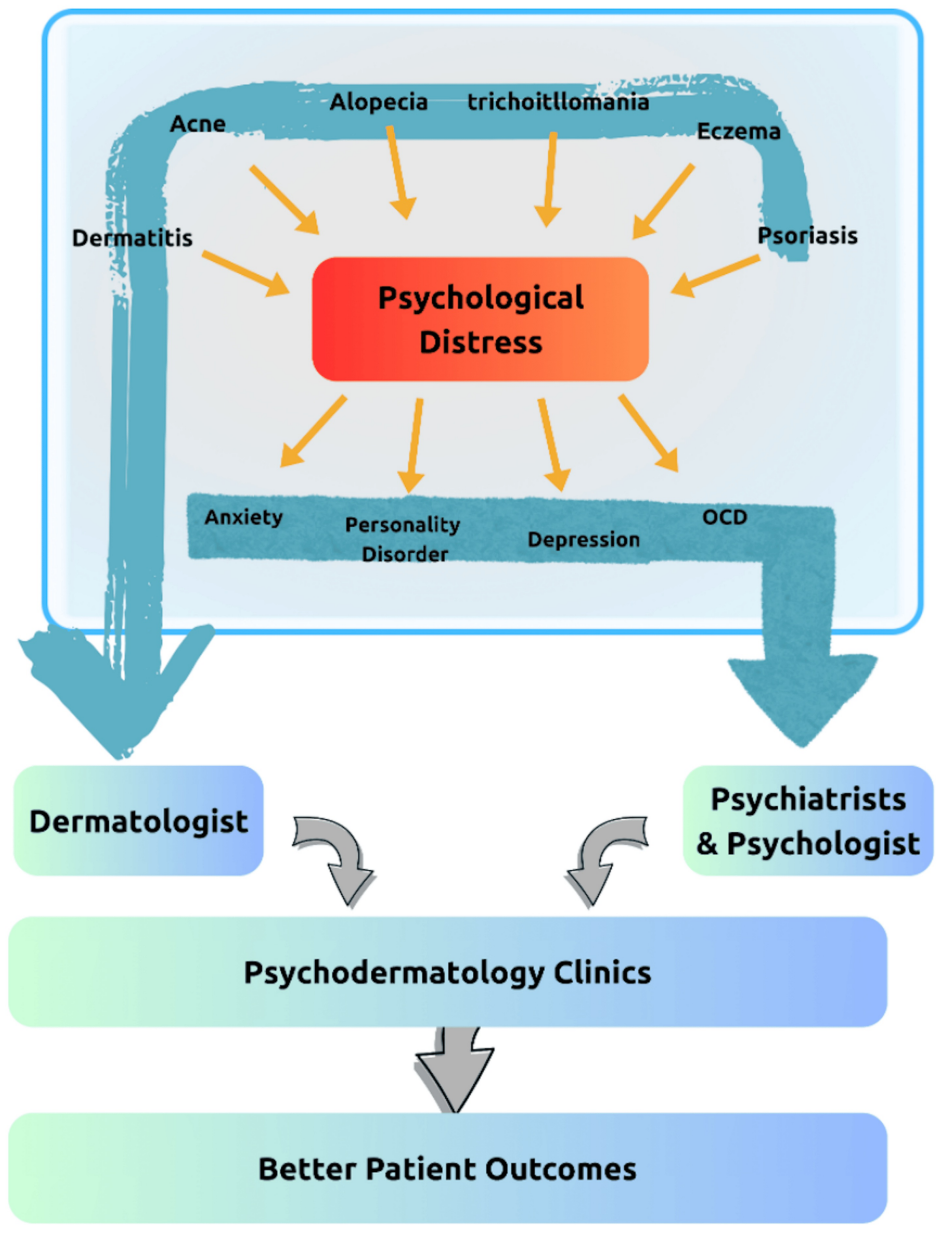
Collaboration Between Dermatology and Psychiatry Image credit: [35,45,47,48], created via Canva (Canva Pty Ltd., Sydney, Australia) by Ansa Bakhtawar.

Despite growing evidence, psychodermatology is not yet widely implemented. Barriers include a lack of trained mental health professionals in dermatology, limited referral pathways, time constraints, and persistent stigma surrounding psychiatric illness. Structured screening protocols, formal referral systems, and psychodermatology training in residency curricula could help bridge these care gaps [38,45]. Early psychotherapeutic intervention can improve adherence, reduce maladaptive behaviors such as scratching, and address the compounded psychosocial burden faced by patients with inflammatory and pigmentary/scarring dermatoses [11,37,46]. Figure 4 illustrates the relationship among dermatologists, psychologists, and psychiatrists.

Psychosocial impacts and adjunctive role of PRP in improving outcomes of CSC in young adults

CSCs are highly prevalent among young people, with acne vulgaris affecting up to 85% of the population, mostly adolescents [49]. These disorders often emerge during a critical stage of psychosocial development, leading to low self-esteem, social withdrawal, anxiety, and depression [50]. This psychological distress may, in turn, exacerbate skin disease. In acne, for instance, stress-induced activation of CRH receptors on sebocytes increases sebum production and pro-inflammatory cytokines [51]. Stress has also been linked to skin barrier dysfunction and the perpetuation of itch-scratch cycles in conditions like atopic dermatitis [52].

**Figure 5:**
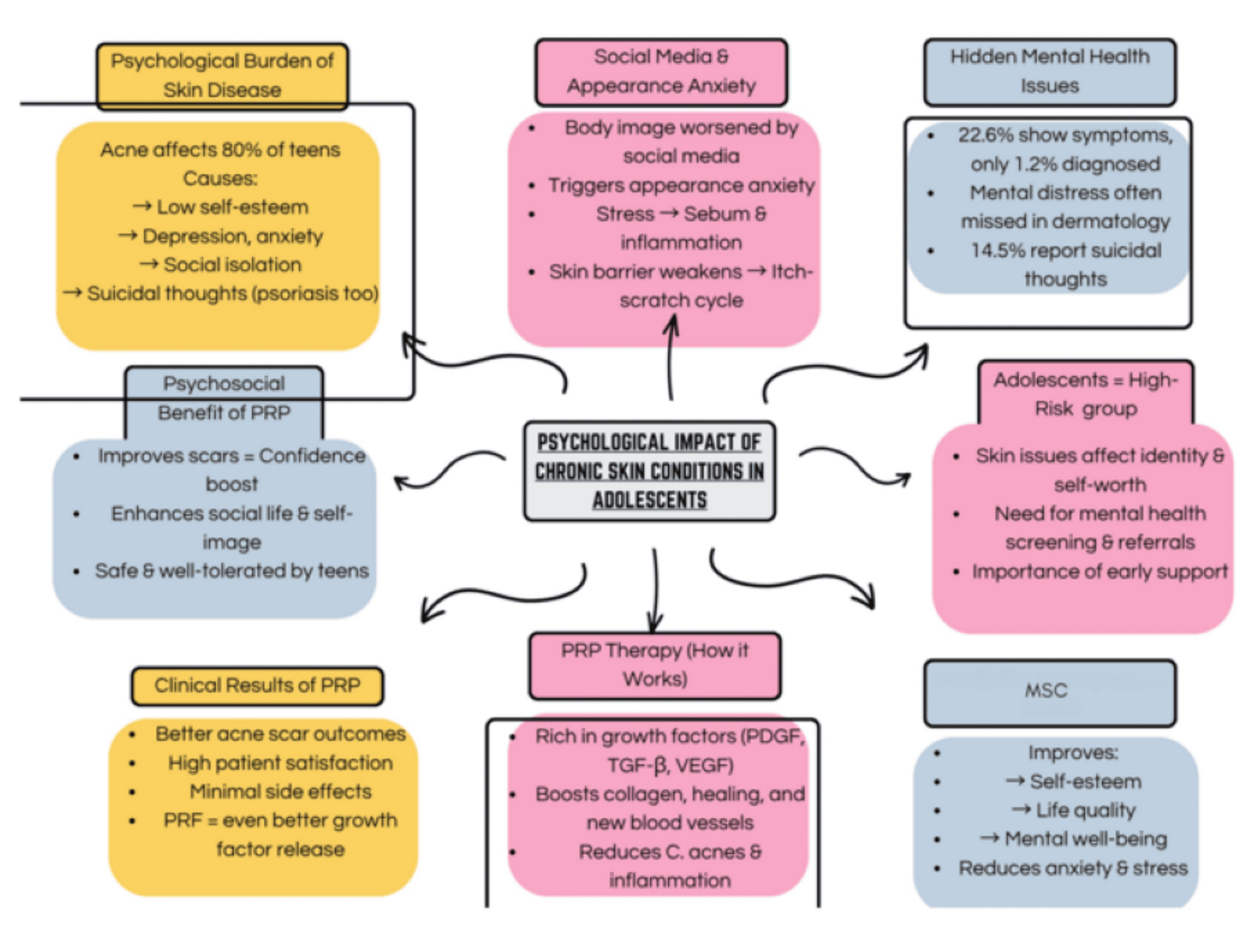
Psychological Impact of Chronic Skin Conditions in Adolescents Image credit: [11,49-55], created via Canva (Canva Pty Ltd., Sydney, Australia) by Isra Meraj. PRP: Platelet-Rich Plasma; PDGF: Platelet-Derived Growth Factor; TGF-β: Transforming Growth Factor Beta; VEGF: Vascular Endothelial Growth Factor; MSC: Mindful Self-Compassion

Despite this burden, under-recognition of psychosocial distress remains a major concern. A meta-analysis found that while 22.6% of young people with CSC report mental health symptoms, only 1.2% receive a formal diagnosis [11]. Barriers include patient reluctance, limited consultation time, and clinician misattribution of affective symptoms to skin disease [53,54]. Alarmingly, suicidal ideation or behavior ranges from 0.45% in psoriasis to 67% in BDD [55]. In acne, suicide attempt rates reach up to 21.9%, highlighting the urgent need for structured psychosocial screening [55]. Dermatology services should integrate validated mental health tools, establish referral pathways, and provide non-stigmatizing, psychosocial support as part of care. Figure 5 illustrates the impact of CSCs on adolescent mental health.

In parallel, PRP is increasingly sought as an adjunctive therapy for acne and scarring. PRP may exert anti-inflammatory and antimicrobial effects by modulating local immune responses and potentially suppressing *Cutibacterium acnes* colonization [56]. A split-face study comparing microneedling plus topical PRP with microneedling alone, after subcision, reported superior scar improvement and patient satisfaction, with minimal adverse events [57]. These findings align with earlier work [58] showing greater acne severity reduction when PRP was combined with microneedling.

Comparisons between PRP and PRF, a second-generation autologous concentrate without anticoagulants, suggest that PRF has higher cumulative growth factor release and more pronounced improvements, particularly when combined with microneedling [8]. Given that scarring can be a long-term source of psychological distress, PRP’s ability to deliver visible cosmetic gains, with minimal downtime, holds particular relevance in adolescents. Its favorable safety profile, with low rates of serious adverse events, further supports its use as an adjunct in this population [57].

Psychosocial interventions in dermatology

A growing body of evidence supports the role of non-pharmacological psychosocial interventions in improving both dermatological outcomes and mental well-being in CSCs such as psoriasis, eczema, and AA. Multiple psychotherapeutic modalities, including CBT, mindfulness-based interventions, hypnotherapy, and stress-reduction techniques, have demonstrated improvements in both psychological and dermatological outcomes [40,59,60].

CBT

CBT is a structured, goal-oriented approach that identifies and challenges maladaptive thoughts, while promoting adaptive cognitive and behavioral patterns [37]. In dermatology, its primary objective is to strengthen coping skills, reduce pessimistic thought patterns, and enhance psychological resilience [46]. CBT is particularly effective in conditions precipitated or exacerbated by psychological stress, reducing anxiety and depression [37]. However, integration into dermatological practice is limited by the few dermatologists trained in CBT and by restricted access to appropriate psychological services [37].

CBT has been associated with improvements in anxiety, depression, and QoL, even without concurrent visible skin improvement [36,37]. Combining CBT with standard treatments, such as NB-UVB phototherapy, enhances clinical response, with PASI75 rates rising from 15% (NB-UVB alone) to 65% (CBT + NB-UVB) [37]. Self-guided CBT programs - brief, interactive weekly sessions with remote therapist support - offer lower-cost, accessible alternatives [46].

In hair disorders, mindfulness-based stress reduction (MBSR) improved QoL, reduced relationship strain, and alleviated anxiety, phobia, and distress in AA and scarring alopecia [60]. Evidence for CBT in AA remains preliminary but promising [37].

Mindfulness-Based Approaches

MBSR and mindful self-compassion (MSC) reduce psychological distress and enhance self-acceptance. MBSR cultivates non-judgmental awareness of present-moment experiences to reduce emotional reactivity, while MSC fosters self-kindness and acceptance during distress. Online MSC/MBSR training, combined with usual management for adults with atopic dermatitis, improved QoL and symptom control, although external validity was limited by self-selection bias [61]. Four weeks of online MSC significantly improved QoL, self-esteem, and emotional resilience in individuals with chronic skin disease [34]. A systematic review found that MBSR improved anxiety, distress, and relationship impacts in AA, although high-quality CBT trials in this population remain limited [60].

Adjunctive Behavioral and Stress-Reduction Techniques

Psychodermatologic stress-reduction interventions, such as autogenic training (AT), progressive muscle relaxation (PMR), habit-reversal training (HRT), hypnotherapy, music therapy, and massage, reduce the psychophysiological burden of skin disease, improve adherence, and mitigate maladaptive behaviors like scratching [37]. In acne, psychotherapeutic stress-reduction interventions have been associated with reductions in both psychiatric symptoms and skin lesion severity, paralleling improvements seen with oral isotretinoin therapy [36].

AT promotes focused attention on body areas, with positive self-suggestions to relieve symptoms [40]. PMR systematically tenses and relaxes muscle groups to reduce physical and psychological arousal [40]. HRT replaces maladaptive behaviors with alternative coping responses, such as using a stress-relief object during pruritic flare triggers (e.g., squeezing a ball to prevent scratching) [40].

Hypnotherapy uses guided induction into a trance-like state to facilitate acceptance of therapeutic suggestions at a subconscious level. In eczema, this might include visualizing the skin as “cool and comfortable.” In eczema, 10 hypnotherapy sessions increased cutaneous pain thresholds in patients compared with healthy controls [40]. Hypnotherapy in adults reduced symptoms and steroid use, while in children, a “magic music” relaxation recording improved eczema activity, itching, sleep quality, and mood for at least two months post-intervention [40].

Music therapy, particularly the “U sequence” technique, delivers music that gradually slows and maintains a steady rhythm, and then accelerates, in a quiet, darkened setting for around 20 minutes [40]. In a controlled trial, 64% of patients with chronic pruritic dermatoses (mainly eczema) experienced reduced itch following music therapy, compared with emollient application alone; 91% recommended the approach [40].

**Figure 6:**
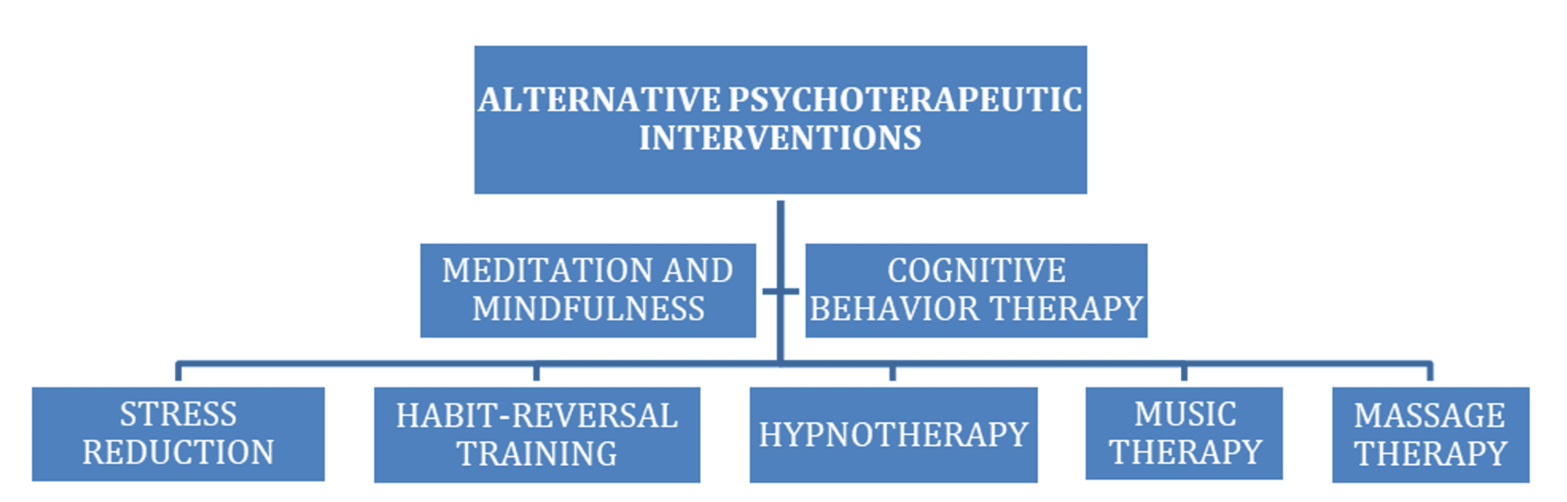
Alternative Psychotherapeutic Interventions This figure has been made to give an illustrative overview; hence, it highlights selected alternative psychotherapeutic interventions relevant to psychodermatology. Image credit: [34,40,59,61], created via Microsoft Word (Microsoft® Corp., Redmond, WA, USA) by Paola Chavez.

Massage therapy, administered alongside standard care in children with eczema, significantly improved erythema, lichenification, scaling, excoriation, itch, anxiety, mood, and coping skills [40]. Figure 6 highlights alternative options for psychotherapeutic interventions.

Research gaps and future directions

Future research should prioritize high-quality RCTs with standardized PRP protocols to improve reproducibility and comparability [62]. Expanding PRP’s indications across diverse dermatological conditions also warrants investigation.

In psychodermatology, there is a need for validated screening tools tailored to age, diagnosis, and cultural context [11]. Awareness among primary care physicians about the psychological burden of skin disease contributes to underdiagnosis and undertreatment and should be addressed. Access to interventions such as CBT is restricted by a shortage of trained dermatologists, inadequate referral pathways, and insufficient collaboration with mental health professionals [36]. Routine mental health screening at dermatology visits could improve early detection and longitudinal monitoring. Multidisciplinary care models, integrating dermatology, psychiatry, and psychology, can enhance both physical outcomes and psychosocial well-being [36].

A major evidence gap is the lack of studies combining PRP with psychological interventions. This integrative approach could offer synergistic benefits, addressing biological regeneration and mental health together. Future trials should examine whether PRP, when paired with interventions such as CBT, improves symptoms, self-esteem, and treatment adherence in chronic skin disease.

Challenges and limitations

Current PRP research is limited by substantial methodological variability. Heterogeneity in preparation - including centrifugation speed, time, platelet concentration, and administration protocols - remains the major barrier to clinical translation. Many studies are small, retrospective, or observational, introducing selection bias and reducing the strength of evidence. Long-term follow-up is scarce, hindering evaluation of treatment durability. Patient-specific factors, such as obesity, diabetes, and underlying stress, which influence regenerative efficacy, are often underreported or uncontrolled.

This review has been restricted to English-language articles published between 2015 and 2025, introducing potential selection bias and excluding earlier or non-English studies. PRISMA methodology was not applied, and inclusion relied on reviewer judgment, limiting reproducibility.

In psychodermatology, research often relies on self-reported psychological outcomes without validated tools, and no widely adopted screening instruments exist for routine practice.

## Conclusions

PRP is a versatile regenerative therapy in dermatology, demonstrating benefits in acne, scarring, melasma, alopecia, vitiligo, and chronic wounds through growth factor-mediated tissue repair, with a favorable safety profile. Psychodermatology complements these benefits by addressing the psychological burden of skin disease, thereby improving adherence and enhancing overall outcomes. Dermatologists should actively facilitate access to psychological support, particularly in conditions with high psychosocial impact. To date, no studies have evaluated the combined use of PRP and psychodermatologic interventions. Future research should investigate integrated regenerative-psychological treatment models to deliver holistic, personalized care. Broader access to trained practitioners and high-quality RCTs remain essential to optimize implementation. By uniting biological regeneration with psychological support, dermatology can advance toward truly patient-centered care, treating not only the skin but the patient as a whole.
